# Giant barocaloric effects at low pressure in ferrielectric ammonium sulphate

**DOI:** 10.1038/ncomms9801

**Published:** 2015-11-26

**Authors:** P. Lloveras, E. Stern-Taulats, M. Barrio, J.-Ll. Tamarit, S. Crossley, W. Li, V. Pomjakushin, A. Planes, Ll. Mañosa, N. D. Mathur, X. Moya

**Affiliations:** 1 Departament de Física i Enginyeria Nuclear, ETSEIB, Universitat Politècnica de Catalunya, Diagonal 647, Barcelona, 08028 Catalonia, Spain; 2 Facultat de Física, Departament d'Estructura i Constituents de la Matèria, Universitat de Barcelona, Martí i Franquès 1, Barcelona, 08028 Catalonia, Spain; 3 Department of Materials Science, University of Cambridge, 27 Charles Babbage Road, Cambridge CB3 0FS, UK; 4 School of Physics and Wuhan National High Magnetic Field Center, Huazhong University of Science and Technology, Wuhan 430074, China; 5 Laboratory for Neutron Scattering and Imaging, Paul Scherrer Institut (PSI), CH-5232 Villigen PSI, Switzerland

## Abstract

Caloric effects are currently under intense study due to the prospect of environment-friendly cooling applications. Most of the research is centred on large magnetocaloric effects and large electrocaloric effects, but the former require large magnetic fields that are challenging to generate economically and the latter require large electric fields that can only be applied without breakdown in thin samples. Here we use small changes in hydrostatic pressure to drive giant inverse barocaloric effects near the ferrielectric phase transition in ammonium sulphate. We find barocaloric effects and strengths that exceed those previously observed near magnetostructural phase transitions in magnetic materials. Our findings should therefore inspire the discovery of giant barocaloric effects in a wide range of unexplored ferroelectric materials, ultimately leading to barocaloric cooling devices.

## Abstract (web summary)

Large barocaloric effects driven by pressure may lead to environmentally friendly cooling, but they have only been observed in a small number of relatively expensive magnetic materials. Here, the authors show large barocaloric effects near the ferrielectric phase transition in ammonium sulphate.

## Graphical abstract

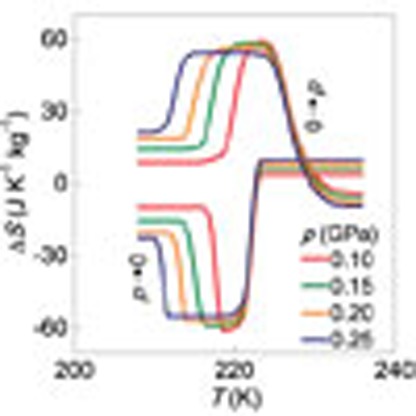

Foodstuffs, beverages, medicine, electronics and populated spaces all require cooling, but existing refrigeration and air-conditioning units rely primarily on the compression and expansion of environmentally harmful fluids. Resurgent interest in solid materials that display magnetically, electrically and mechanically driven phase transitions near room temperature^1^,^2^,^3^ has provoked interest in the possibility of environment-friendly cooling applications, but these will only come to fruition if it is possible to develop or discover inexpensive materials that show large reversible thermal changes in response to fields that are small and easy to generate.

Mechanical stress is easy to generate, but large barocaloric (BC) effects driven by hydrostatic pressure near phase transitions have only been observed in a small number of relatively expensive magnetic materials, where changes of magnetization are accompanied by changes in crystal symmetry^4^,^5^ or volume alone^6^,^7^,^8^ (Table 1). (Large BC effects have also been observed in poly(methyl methacrylate) away from any transition^9^.) Here we demonstrate giant BC effects near the ferrielectric phase transition^10^,^11^,^12^,^13^ in a powder of ammonium sulphate (AS) [(NH_4_)_2_SO_4_], which is made from cheap abundant elements and enjoys widespread agricultural use as a fertilizer. We use calorimetry to identify pressure-driven isothermal entropy changes of |Δ*S*|∼60 J K^−1^ kg^−1^, which exceed the corresponding values that have been found for metallic alloys near first-order magnetic phase transitions (∼10–25 J K^−1^ kg^−1^; Table 1), and predicted for PbTiO_3_ and BaTiO_3_ near first-order ferroelectric phase transitions^14^,^15^ (∼3–4 J K^−1^ kg^−1^). These giant entropy changes are driven using small changes of hydrostatic pressure |Δ*p*|=|*p*−*p*_atm_|∼|*p*|∼0.1 GPa, yielding giant BC strengths^1^ |Δ*S*|/|Δ*p*|, |*Q*|/|Δ*p*| and |Δ*T*|/|Δ*p*| (Table 1) (where *Q* is the heat, *T* is the temperature and atmospheric pressure *p*_atm_∼0 GPa). Our giant BC effects may be understood via pressure-driven changes in ionic ordering, whereas the smaller BC effects in magnetic materials^4^,^5^,^6^,^7^,^8^ arise due to pressure-driven changes in the density of electronic states near the Fermi level.

## Results

### Ferrielectric phase transition in AS at atmospheric pressure

**Figure 1:**
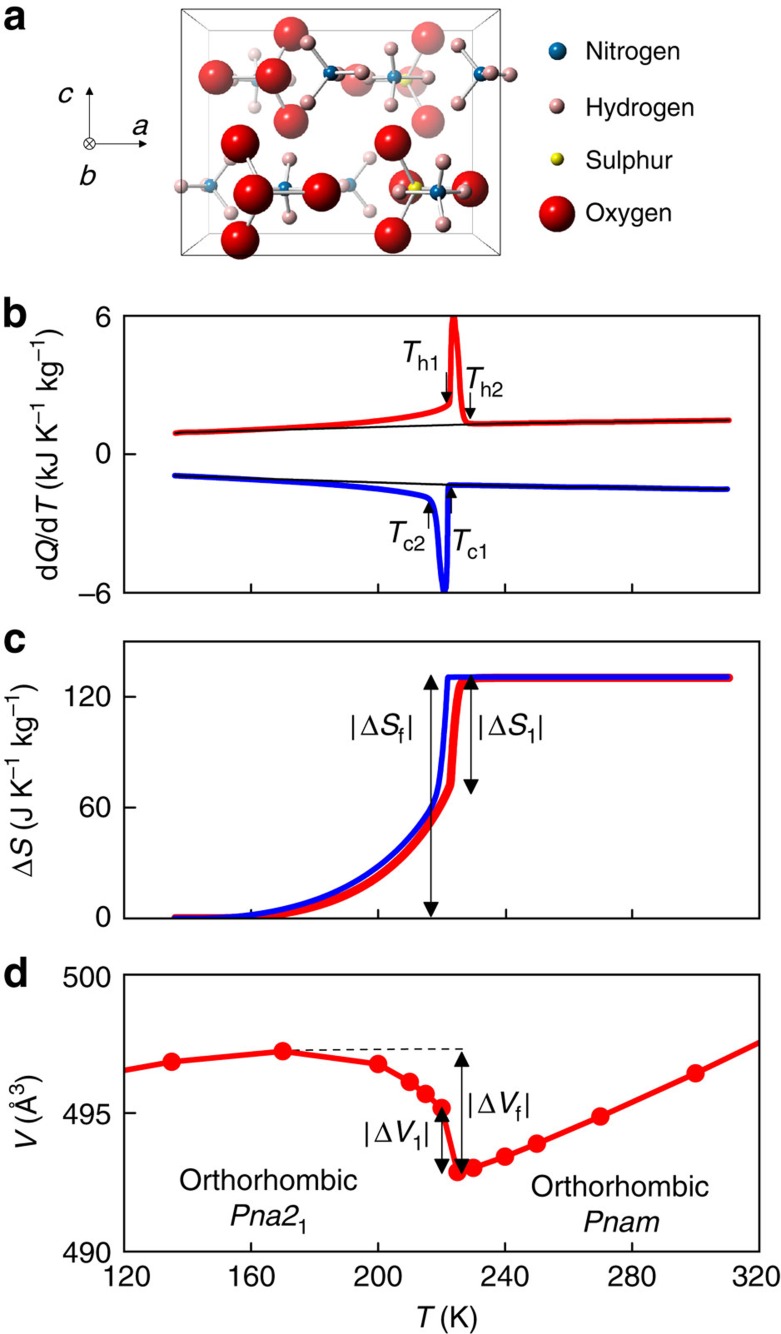
Ferrielectric phase transition in AS at atmospheric pressure. (**a**) Unit cell of the high-temperature orthorhombic phase (*Pnam*). (**b**) Heat flow d*Q*/d*T* on cooling (blue) and heating (red) across the full transition. Baselines are black and d*Q*/d*T*>0 denotes endothermic processes. (**c**) Resulting entropy change Δ*S*(*T*) with respect to the low-temperature phase, revealing entropy changes for the first-order transition (|Δ*S*_1_|) and the entire transition (|Δ*S*_f_|). (**d**) Unit-cell volume *V*(*T*) on heating, revealing volume changes for the first-order transition (|Δ*V*_1_|) and the entire transition (|Δ*V*_f_|).

At room temperature, AS adopts a centrosymmetric orthorhombic structure (*Pnam*) with four formula units per unit cell comprising three ionic groups (Fig. 1a) that are understood to adopt a disordered configuration at any given instant^16^,^17^. On cooling, the material is generally considered to undergo a reversible order–disorder phase transition to an orthorhombic polar structure (*Pna*2_1_) that is ferrielectric^10^,^11^. Our heat flow d*Q/*d*T* measurements confirm that this transition occurs in two steps^10^,^11^,^12^,^13^. First, the symmetry change arises from a non-isochoric first-order transition at *T*_1_∼221 K associated with partial ionic ordering (Fig. 1b). Second, further ordering yields additional changes of volume in a continuous manner down to ∼160 K (Fig. 1b-d). (Figure 1d was obtained using temperature-dependent lattice parameters (Supplementary Fig. 1) calculated from X-ray diffraction patterns (Supplementary Fig. 2).) The first-order transition is weakly hysteretic and occurs at *T*_1_∼224 K on heating. Its start and finish temperatures on cooling are *T*_c1_∼223 K and *T*_c2_∼216 K, respectively, and its start and finish temperatures on heating are *T*_h1_∼222 K and *T*_h2_∼229 K, respectively.

Integration of (d*Q/*d*T*)/*T* yields the corresponding entropy change Δ*S*(*T*) (Fig. 1c), with |Δ*S*_f_|=130±6 J K^−1^ kg^−1^ for the full transition. Integration of d*Q/*d*T* across the full transition yields a corresponding heat of |*Q*_f_|=29±2 kJ kg^−1^. These values are in good agreement with previous experimental values^12^,^13^ of |Δ*S*_f_|∼126–133 J K^−1^ kg^−1^ and |*Q*_f_|∼28–30 kJ kg^−1^, and are consistent with the change of entropy |Δ*S*_f_|=3*R*ln2=130 J K^−1^ kg^−1^ expected^16^ for an order-disorder transition involving three ionic groups per formula unit (*R*=8.314 J K^−1^ mol^−1^). For the first-order transition alone, integration yields |Δ*S*_1_|=65±4 J K^−1^ kg^−1^ and latent heat |*Q*_1_|=14.5±1.0 kJ kg^−1^. These values correspond to ∼50% of the aforementioned values for the full transition and closely match literature values^13^ of |Δ*S*_1_|=61 J K^−1^ kg^−1^ and |*Q*_1_|=13.6 kJ kg^−1^ for deuterated AS [(ND_4_)_2_SO_4_], where no aspect of the transition is modified by the deuteration.

On heating through the ferrielectric transition, X-ray diffraction data confirm the expected changes in crystal structure^10^,^11^,^13^. The unit-cell volume *V* decreases by ∼0.9% across the full transition (Δ*V*_f_=−4.4±0.2 Å^3^) and by ∼0.5% across the first-order transition alone (Δ*V*_1_=−2.5±0.2 Å^3^) (Fig. 1d). Given that BC effects per unit mass *m* due to pressure change Δ*p*=*p*_2_−*p*_1_ may be expressed using the Maxwell relation *m*^−1^(∂*V*/∂*T*)_*p*_=−(∂*S*/∂*p*)_*T*_ as^1^ Δ*S*(*p*_1_→*p*_2_)=−*m*^−1^
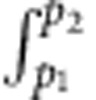
(∂*V*/∂*T*)_*p*′_d*p*′, we anticipate inverse BC effects in the transition regime where (∂*V*/∂*T*)_*p*=0_<0 and we anticipate conventional BC effects away from the transition regime where (∂*V*/∂*T*)_*p*=0_>0.

### Ferrielectric phase transition in AS under applied pressure

**Figure 2:**
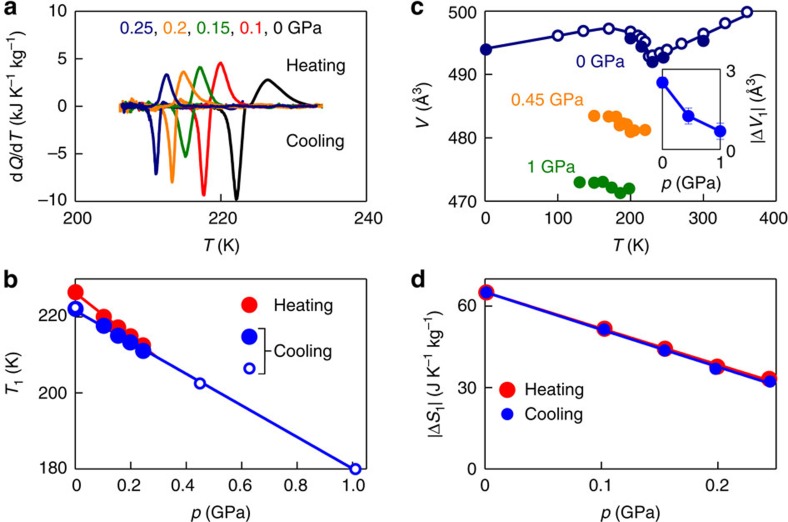
Ferrielectric phase transition in AS under applied pressure. (**a**) Heat flow d*Q*/d*T* on cooling and heating across the transition for different values of increasing pressure *p*, after baseline subtraction. (**b**) Transition temperature *T*_1_(*p*) for the first-order transition, obtained below 0.3 GPa from the calorimetric data of **a** (closed symbols) and below ∼1.0 GPa from neutron diffraction of deuterated AS (open symbols). (**c**) Unit-cell volume *V*(*T*) obtained on cooling at selected pressures from neutron diffraction of deuterated AS (closed symbols), with inset showing |Δ*V*_1_(*p*)|. The X-ray diffraction data of Fig. 1d are included to demonstrate consistency (open symbols). (**d**) Entropy change |Δ*S*_1_(*p*)| for the first-order transition, obtained from the calorimetric data of **a**. Lines in **b** and **d** represent linear fits.

For the first-order transition, heat flow measurements d*Q*/d*T* reveal a strong pressure-induced shift in *T*_1_ (Fig. 2a,b), with d*T*_1_/d*p*=−57±4 K GPa^−1^ on heating and d*T*_1_/d*p*=−45±4 K GPa^−1^ on cooling. A similar shift of −45±6 K GPa^−1^ on heating is obtained via the Clausius-Clapeyron equation d*T*_1_/d*p*=Δ*v*_1_/Δ*S*_1_, using Δ*S*_1_=65±4 J K^−1^ kg^−1^ (Fig. 1c) and specific volume change Δ*v*_1_=−(2.9±0.2) × 10^−6^ m^3^ kg^−1^ (from Fig. 1d). These large values of d*T*_1_/d*p* are similar to those reported for single-crystal AS^18^,^19^ and magnetic alloys (Table 2), and indicate that the narrow first-order transition of width *T*_c1_−*T*_c2_∼*T*_h2_−*T*_h1_∼7 K may be fully driven in either direction using moderate values of |Δ*p*|∼0.15 GPa.

The discrepancy in values of *T*_1_(*p*) measured on heating and cooling (Fig. 2b) evidences a thermal hysteresis that is suppressed below the maximum value of *p*∼0.3 GPa for our calorimeter. At even higher pressures, neutron diffraction data for deuterated AS reveal that d*T*_1_/d*p* remains constant (open symbols, Fig. 2b), while |Δ*V*_1_| falls (Fig. 2c), implying via the Clausius–Clapeyron equation a pressure-induced suppression of |Δ*S*_1_|. (Figure 2c was obtained using temperature-dependent lattice parameters (Supplementary Fig. 1) calculated from neutron diffraction patterns (Supplementary Fig. 3).) This suppression was confirmed (Fig. 2d) from finite-pressure plots of |Δ*S*_1_(*T*)| (Supplementary Fig. 4a,b) obtained from the calorimetric data of Fig. 2a, as described in Methods.

### BC effects in AS

The fall in |Δ*S*_1_(*p*)| arises because of additional changes in isothermal entropy Δ*S*_+_(*p*) that are reversible, large and change sign across the first-order transition. Above *T*_1_(*p*), these additional entropy changes correspond to conventional BC effects associated with elastic heat, which arises at all temperatures, except while driving transitions. Near and below *T*_1_(*p*), these additional entropy changes correspond to inverse BC effects, because the continuous part of the full transition precludes elastic heat. The additional entropy changes would be challenging to detect via the calorimetry of Fig. 2, but they may be expressed^1^ away from the first-order transition as Δ*S*(*p*_1_→*p*_2_)=−*m*^−1^
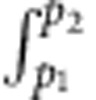
(∂*V*/∂*T*)_*p*′_d*p*′, using the aforementioned Maxwell relation with *S*_+_ replacing *S*. From this formulation, we anticipate large values of Δ*S*_+_ given an AS volumetric thermal expansion coefficient *V*^−1^(∂*V*/∂*T*)_*p*_ whose magnitude ∼10^−4^ K^−1^ (Supplementary Fig. 5a) exceeds the corresponding values^6^,^20^,^21^,^22^,^23^,^24^ of ∼10^−7^–10^−5^ K^−1^ for the magnetic BC materials of Table 1.

**Figure 3:**
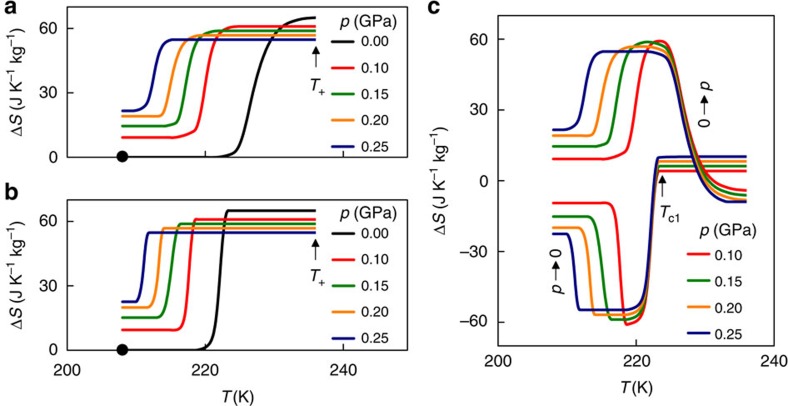
Giant inverse BC effects in AS. (**a**,**b**) Entropy change Δ*S*(*T*,*p*) with respect to *S*(*T*=208 K, *p*=0) (black dot), deduced using additional entropy change Δ*S*_+_(*p*) at *T*_+_=236 K, to offset the pressure-dependent entropy change Δ*S*_1_(*T*) that arises on (**a**) heating and (**b**) cooling through the first-order transition. (**c**) Isothermal entropy change Δ*S* for increasing pressure (0→*p*) as deduced from **a** and for decreasing pressure (*p*→0) as deduced from **b**. Reversibility is apparent up to a few degrees below *T*_c1_(*p*=0)∼223.5 K.

To confirm that the fall in |Δ*S*_1_(*p*)| arises due to additional changes of entropy Δ*S*_+_ away from *T*_1_(*p*), we evaluated Δ*S*_+_(*p*) on applying pressure above *T*_1_(*p*) at *T*_+_=236 K by assuming (∂*V*/∂*T*)_*p*_ to be independent of pressure such that Δ*S*_+_(*p*)=−[*m*^−1^(∂*V*/∂*T*)_*p*=0_]*p* (pressure-dependent data are unavailable due to inaccurate low-pressure control coupled with excessive neutron acquisition times). Choosing *T*_+_>*T*_1_(*p*) is convenient, because it avoids the forbidden possibility of *T*_1_(*p*) falling to *T*_+_ at high pressure. Using the resulting values of Δ*S*_+_(*p*) at *T*_+_=236 K (Supplementary Fig. 5b) to displace at this temperature the finite-pressure plots of Δ*S*_1_(*T*) (Supplementary Fig. 4a,b for heating and cooling, respectively), we have constructed finite-pressure plots of total entropy change Δ*S*(*T*,*p*) (Fig. 3a,b) specified with respect to the zero-pressure total entropy below the first-order transition at 208 K. Whether the calorimetrically accessible value of Δ*S*_1_(*T*) was measured on heating (for Fig. 3a) or cooling (for Fig. 3b), the resulting values of Δ*S*(208 K, *p*) match well with predictions of Δ*S*_+_(*p*) that were obtained by setting *T*_+_ to 208 K (Supplementary Fig. 5b), thus providing quantitative confirmation that the fall in |Δ*S*_1_(*p*)| arises due to the sign change in BC effects across the first-order transition.

**Figure 4:**
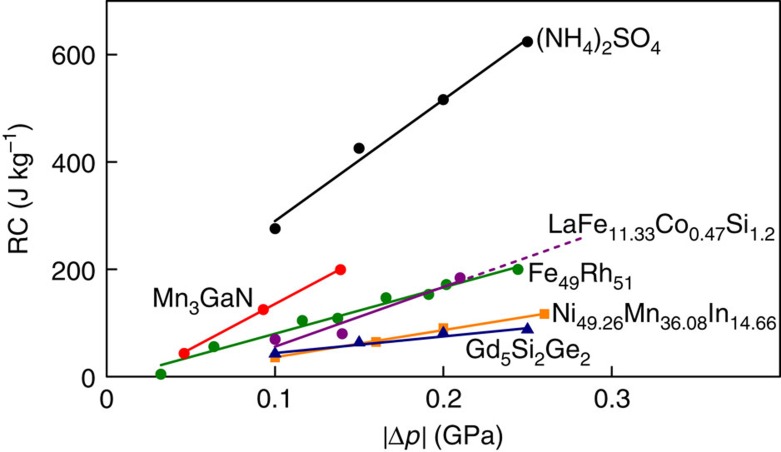
Refrigerant capacity RC for giant BC materials. For the materials in Table 1, we compare values of RC=|Δ*S*| × (FWHM of Δ*S*(*T*)) for selected pressure changes of magnitude |Δ*p*|=|*p*−*p*_atm_|∼|*p*|, using Δ*S*(*T*) for BC cooling, and constraining *T* to ensure reversibility (except for LaFe_11.33_Co_0.47_Si_1.2_, where only BC cooling data are available, and Ni_49.26_Mn_36.08_In_14.66_, where only BC heating data are available). Solid lines represent linear fits.

Our plot of Δ*S*(*T*,*p*) for data obtained on heating (Fig. 3a) permits us to establish isothermal BC effects on applying pressure (Fig. 3c), as heating and high pressure both tend to favour the high-temperature, high-pressure centrosymmetric phase. Similarly, our plot of Δ*S*(*T*,*p*) for data obtained on cooling (Fig. 3b) permits us to establish isothermal BC effects on decreasing pressure (Fig. 3c), as cooling and low pressure both tend to favour the low-temperature ferrielectric phase. Near and above the value of *T*_c1_(*p*=0) indicated, discrepancies in isothermal entropy change on applying and removing pressure evidence irreversibility. By contrast, reversible BC effects are apparent a few degrees below *T*_c1_(*p*=0) and at all lower temperatures studied, consistent with no significant thermal hysteresis in the first-order transition (Fig. 2b). The largest reversible isothermal entropy change |Δ*S*|∼60±5 J K^−1^ kg^−1^ arises at ∼219 K and exceeds the giant BC effects reported for magnetic alloys (Table 1). The sharpness of the transition in Δ*S*(*T*) (Fig. 3a,b) permits this large entropy change to be achieved with a low value of |Δ*p*|=0.1 GPa (Fig. 3c), yielding giant BC strengths^1^ |Δ*S*|/|Δ*p*| and |*Q*|/|Δ*p*| (Table 1). Larger pressures extend reversible BC effects to lower temperatures, causing the large refrigerant capacity^25^ RC=|Δ*S*| × (FWHM of Δ*S*(*T*)) (Table 1) to increase (Fig. 4) despite the small reduction in |Δ*S*_1_(*p*)| (Fig. 2d) and therefore |Δ*S*(*p*)|. For any given value of applied pressure, AS outperforms all of the magnetic alloys so well that comparable RC values would require much larger changes of pressure (Fig. 4).

Our largest value of |Δ*S*|∼60±5 J K^−1^ kg^−1^, arising due to |Δ*p*|=0.1 GPa at ∼219 K, corresponds to an adiabatic temperature change |Δ*T*|=(*T*/*c*)|Δ*S*|∼8±1 K, using a specific heat capacity *c*=1,700±80 J K^−1^ kg^−1^ (Supplementary Fig. 6) that is assumed to be independent of pressure as usual^4^,^5^,^6^,^7^,^8^. The resulting value of |Δ*T*|/|Δ*p*| is seen to be the largest observed for giant BC materials (Table 1).

## Discussion

Our observation of giant reversible BC effects in ferrielectric salts made from inexpensive abundant elements should inspire the study of BC effects in similar materials, most immediately bulk ferroelectrics that display large thermally driven entropy changes associated with displacive and order-disorder phase transitions. In future, it would be attractive to increase transition temperatures by chemical substitution^26^,^27^ or using an electric field^28^. It would also be attractive to perform direct thermal measurements in the vicinity of room temperature, to confirm the large BC effects predicted using the Maxwell relation (Supplementary Fig. 5b), which are reversible over a wide range of temperatures.

Our findings should stimulate the development of cooling devices based on BC materials, whose energy efficiency^29^,^30^ is good with respect to magnetocaloric, electrocaloric and elastocaloric materials^3^. Unlike elastocaloric materials driven by uniaxial stress, there are no losses or mechanical breakdown associated with plastic deformation. Unlike magnetocaloric materials, there is no need to generate large magnetic fields at great expense. Unlike electrocaloric materials, there is no need to fabricate multilayer devices to exploit giant effects in films^31^. Moreover, the phase transitions giving rise to large BC effects can be driven over a wide range of operating temperatures, unlike both magnetocaloric and electrocaloric materials.

## Methods

### Samples

Powders of AS (≥99.0%) and deuterated AS (≥99.0%) were purchased from Sigma-Aldrich. The typical grain size was <100 μm. AS was used for calorimetry and X-ray diffraction. Deuterated AS was used for neutron diffraction to reduce incoherent scattering.

### Calorimetry at atmospheric pressure

Measurements of heat flow d*Q*/d*T* were performed at atmospheric pressure using a commercial TA Q2000 differential scanning calorimeter at 10 K min^−1^. Heat |*Q*_f_|=|
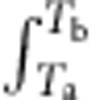
(d*Q*/d*T*′)d*T*′| and entropy change |Δ*S*_f_|=|
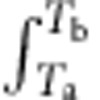
(d*Q*/d*T*′)/*T*′d*T*′| across the full transition were obtained after subtracting baseline backgrounds^32^, with *T*_a_ chosen above (below) the transition on cooling (heating) and *T*_b_ chosen below (above) the transition on cooling (heating). The entropy change on partially driving the transition by heating to temperature *T* is Δ*S*(*T*)=
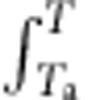
(d*Q*/d*T*′)/*T*′d*T*′. The entropy change on partially driving the transition by cooling to temperature *T* is Δ*S*(*T*)=|Δ*S*_f_|−
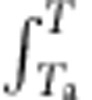
(d*Q*/d*T*′)/*T*′d*T*′.

Zero-field heat capacity data were obtained using the TA Q2000 on cooling in the modulated differential scanning calorimetry mode, with the constant temperature method^33^. The temperature step was 1 K, the temperature modulation was 0.5 K and the period was 60 s.

### Calorimetry under applied pressure

Measurements of heat flow d*Q*/d*T* at constant hydrostatic pressure were performed at ±1–2 K min^−1^, using a differential thermal analyser constructed in-house, with chromel-alumel thermocouples, a Cu–Be Bridgman pressure cell operating up to 0.3 GPa and a circulating thermal bath (Lauda Proline RP 1290, 183–473 K). AS was mixed with an inert perfluorinated liquid and hermetically encapsulated by Sn. DW-Therm (Huber Kältemaschinenbau GmbH) was used as pressure-transmitting medium. For more details, see refs 4, 5, 6, 7. Absolute measurements of temperature in the differential thermal analyser and differential scanning calorimeter differ by ∼1 K.

### X-ray diffraction

High-resolution X-ray diffraction was performed in transmission using Cu *Kα*_1_=1.5406 Å radiation in an INEL diffractometer, with a curved position-sensitive detector (CPS120), a 0.5-mm diameter Lindemann capillary and a 700 series Oxford Cryostream Cooler.

### Neutron diffraction

High-resolution neutron diffraction was performed at the Paul Scherrer Institute, using the high-resolution powder diffractometer for thermal neutrons. Deuterated AS was mixed with NaCl powder to determine the applied pressure, and the mixture was encapsulated in a Pb clamp cell operating up to ∼1 GPa. Temperature was varied using a cryostat operating in 1.4–320 K. The neutron wavelength was set to 1.88570 Å. Lattice parameters were determined by pattern matching using FullProf software.

### Data availability

All relevant data are presented via this publication and Supplementary Information.

## Additional information

**How to cite this article:** Lloveras, P. *et al.* Giant barocaloric effects at low pressure in ferrielectric ammonium sulphate. *Nat. Commun.* 6:8801 doi: 10.1038/ncomms9801 (2015).

## Supplementary Material

Supplementary Information Supplementary Figures 1-6

## Figures and Tables

**Table 1:** Giant BC effects at first-order phase transitions.

**Giant BC material**	***T***	**|Δ*****S*****|**	**|Δ*****T*****|**	**|*****Q*****|**	**|Δ*****p*****|**	**|Δ*****S*****/Δ*****p*****|**	**|Δ*****T*****/Δ*****p*****|**	**|*****Q*****/Δ*****p*****|**	**RC**	**Ref.**
	**K**	**J** **K**^**−1**^ **kg**^**−1**^	**K**	**kJ** **kg**^**−1**^	**GPa**	**J** **K**^**−1**^ **kg**^**−1**^ **GPa**^**−1**^	**K** **GPa**^**−1**^	**kJ** **kg**^**−1**^ **GPa**^**−1**^	**J** **kg**^**−1**^	
Ni_49.26_Mn_36.08_In_14.66_	293	*24*	[4.5]	*7.1*	0.26	92.3	17.3	27.3	120	4
Gd_5_Si_2_Ge_2_	270	*11*	**1.1**	*2.9*	0.20	55	5.5	14.5	81	5
LaFe_11.33_Co_0.47_Si_1.2_	237	*8.7*	**2.2**	*2.0*	0.20	43.5	11	10	180	6
Fe_49_Rh_51_	308	*12.5*	[8.1]	*3.8*	0.11	114	74	34.5	105	7
Mn_3_GaN	285	*21.6*	[4.8]	*6.2*	0.09	232	51.4	66.2	125	8
AS	219	*60*	[8]	*13.2*	0.10	600	80	132	276	This work

BC, barocaloric; |Δ*p*|, hydrostatic pressure change; |*Q*|, isothermal heat; RC, refrigerant capacity; |Δ*S*|, isothermal entropy change; *T*, starting temperature; |Δ*T*|, adiabatic temperature change.

|Δ*S*|, |Δ*T*| and |*Q*| arise at *T*, due to changes of |Δ*p|*. The corresponding strengths |Δ*S*|/|Δ*p*|, |Δ*T*|/|Δ*p*| and |*Q*|/|Δ*p*| were maximized by choosing the smallest values of |Δ*p|* compatible with maximizing |Δ*S*|. Bold entries denote data derived from direct measurements. Italicised entries denote data derived from quasi-direct^1^ measurements. Bracketed entries denote data derived via -*c*Δ*T*≃*T*Δ*S*=*Q* using zero-pressure specific heat capacity *c*. For all entries, *Q*=*T*Δ*S*.

**Table 2:** Properties of first-order phase transitions in giant BC materials.

**Giant BC material**	**|Δ*****V***_**1**_**|**	**|Δ*****V***_**1**_**|/*****V***_**1**_	**|d*****T***_**1**_**/d*****p*****|**	**Ref.**
	**Å**^**3**^	**%**	**K** **GPa**^**−1**^	
Ni_49.26_Mn_36.08_In_14.66_	0.2	0.4	18	4
Gd_5_Si_2_Ge_2_	3.4	0.5	32	5
LaFe_11.33_Co_0.47_Si_1.2_	18	1.2	73	6
Fe_49_Rh_51_	0.3	1.2 (ref. 24)	54	7
Mn_3_GaN	0.6	1.2 (ref. 34)	65	8
AS	2.5±0.2	0.5	45±2	This work

AS, ammonium sulphate; BC, barocaloric; |d*T*_1_/d*p*|, pressure-driven shift in transition temperature; |Δ*V*_1_|, unit-cell volume change; |Δ*V*_1_|/*V*_1_, relative unit-cell volume change.

For AS, we give the shift obtained over a wide pressure range on cooling.
