# Supplementary material for: Giant barocaloric effects at low pressure in ferrielectric ammonium sulphate
Source: Nat Commun. 2015 Nov 26;6:8801. doi: 10.1038/ncomms9801 (PMC4674762; doi:10.1038/ncomms9801)
Supplement: Supplementary Information — Supplementary Figures 1-6 [file ncomms9801-s1.pdf]

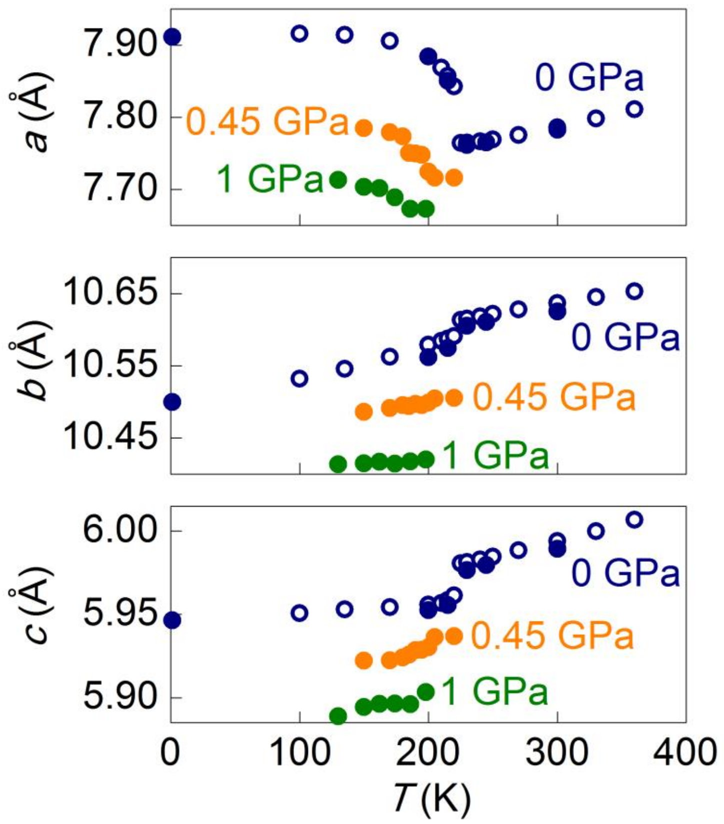

Supplementary Figure 1. Temperature and pressure dependence of AS lattice parameters. Temperature dependence of lattice parameters *a*, *b* and *c* across the structural phase transition at *p* = 0, 0.45 GPa and 1 GPa, obtained from x-ray diffraction on heating AS (open symbols), and neutron diffraction on cooling deuterated AS (closed symbols).

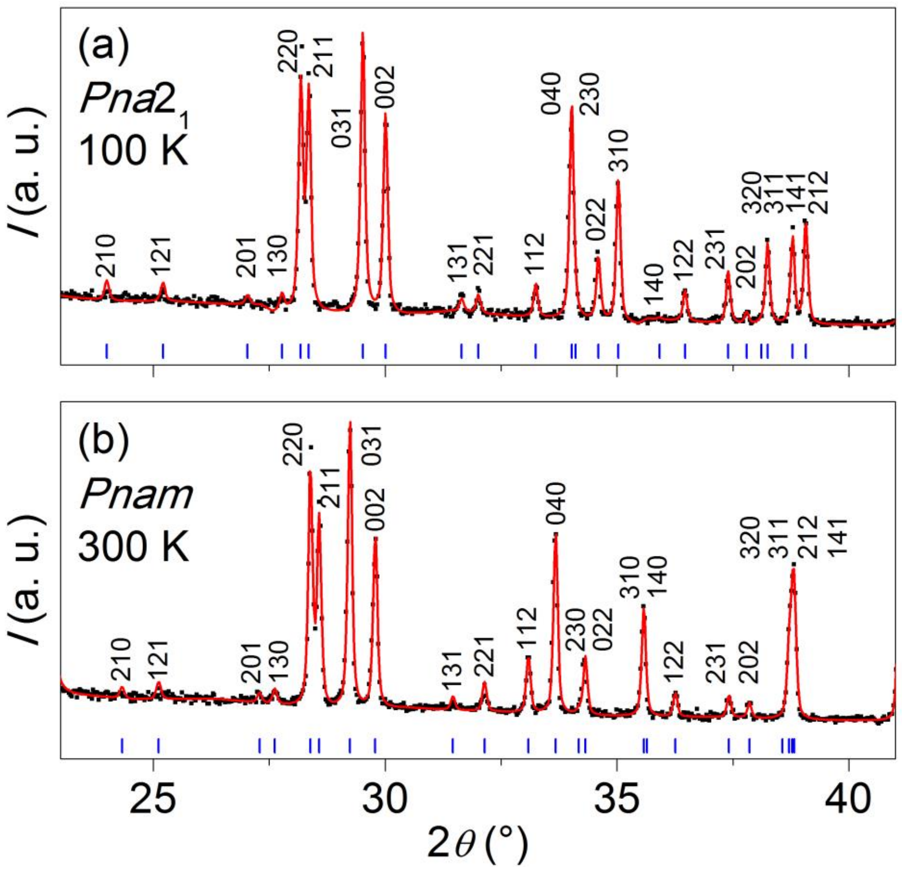

Supplementary Figure 2. X-ray diffraction of AS powder. Detail of selected x-ray diffraction spectra obtained (a) well below and (b) well above the structural phase transition on heating, at atmospheric pressure. Black symbols are experimental data, red lines are fitted patterns, blue lines indicate indexed reflections. The complete dataset is available at https://www.repository.cam.ac.uk/handle/1810/249253.

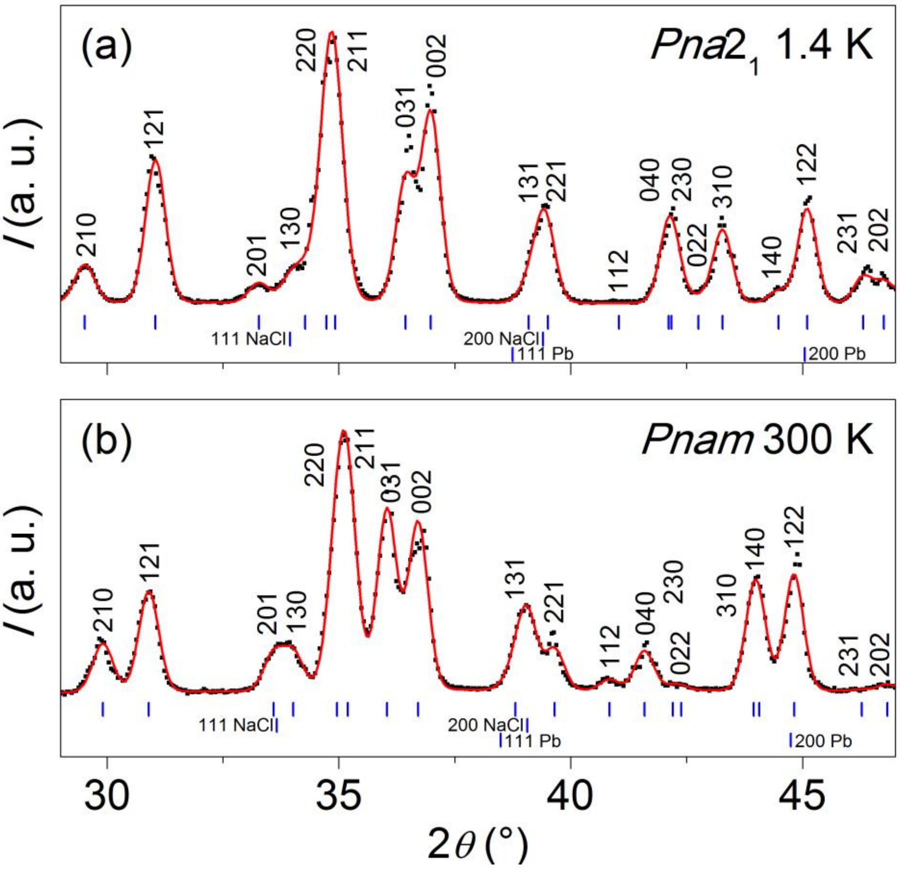

Supplementary Figure 3. Neutron diffraction of deuterated AS powder. Detail of selected neutron diffraction spectra obtained (a) well below and (b) well above the structural phase transition on cooling, at atmospheric pressure. Black symbols are experimental data, red lines are fitted patterns, blue lines indicate indexed reflections. Reflections from NaCl were used to determine the hydrostatic pressure inside the Pb sample holder. The complete dataset is available at https://www.repository.cam.ac.uk/handle/1810/249253.

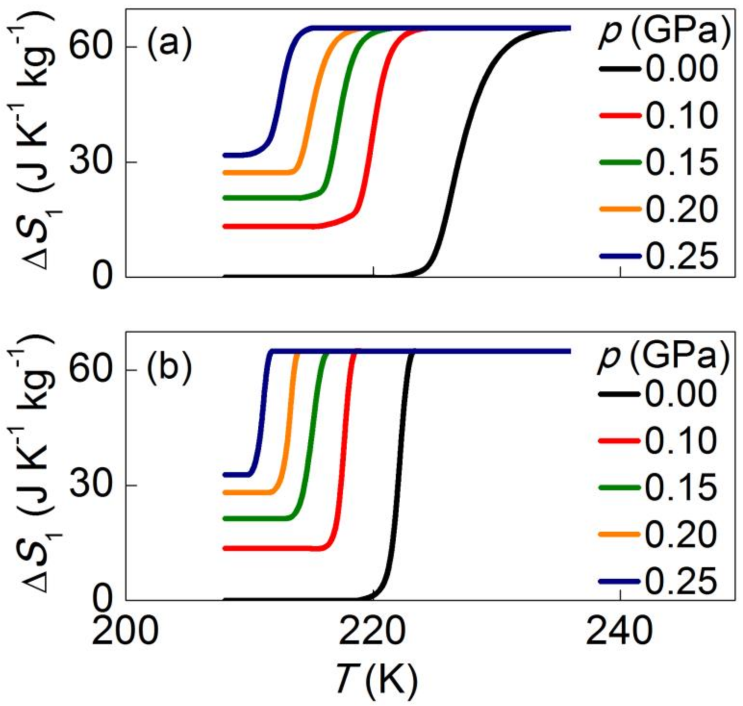

Supplementary Figure 4. Effect of pressure on the entropy change associated with the first-order transition alone. (a,b) Entropy change $\Delta S_1(T,p)$ for the first-order transition obtained by integrating the calorimetric signal [Figure 2(a)] measured on (a) heating and (b) cooling. Values for the high-temperature orthorhombic phase (*Pnam*) are set equal here, and displaced at T_+ = 236 K by $\Delta S_+(p)$ to obtain Figure 3 of the main paper.

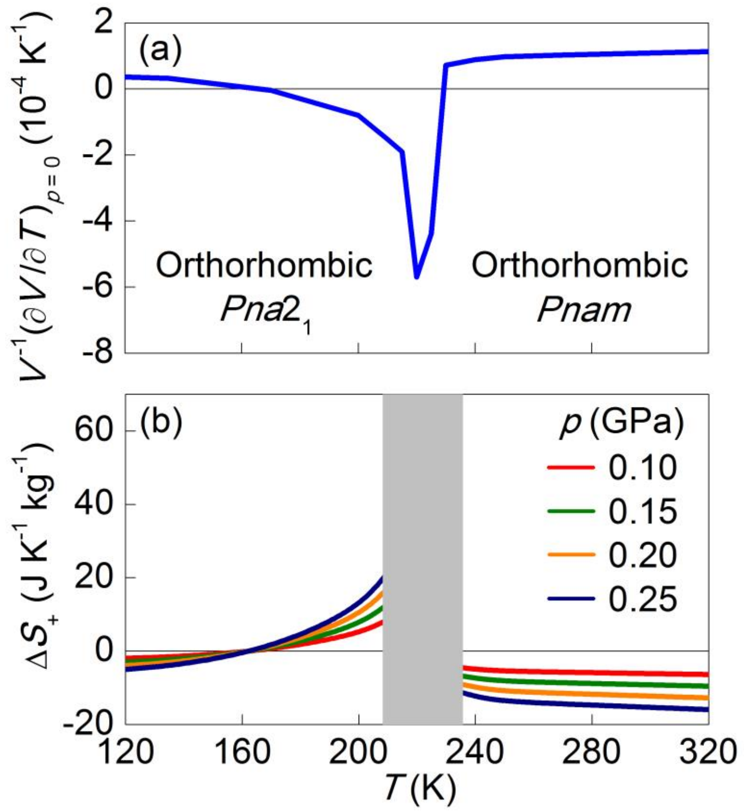

Supplementary Figure 5. Additional entropy change arising reversibly away from the first-order transition. (a) Coefficient of volumetric thermal expansion $V^{-1}(\partial V/\partial T)_{p=0}$ versus temperature, deduced from Figure 1(d). Assuming $(\partial V/\partial T)_p$ to be independent of pressure, we plot $\Delta S_{+}(T,p) = -[m^{-1}(\partial V/\partial T)_{p=0}]p$ on applying pressure p, excluding temperatures (grey zone) in which the pressure-dependent first-order transition lies [Figure S5(a,b)].

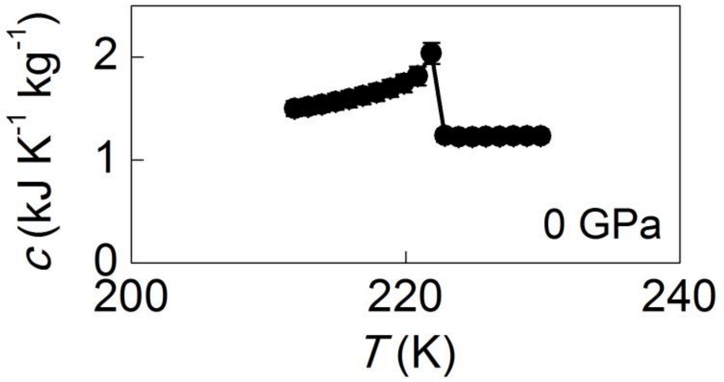

Supplementary Figure 6. Temperature dependence of specific heat capacity. Data measured on cooling in zero pressure.
